# Revisiting the TSH range in older adults: associations between subclinical hypothyroidism and geriatric conditions

**DOI:** 10.1210/jendso/bvag045

**Published:** 2026-02-26

**Authors:** Toshiki Kogai, Hideyoshi Kaga, Toyoyoshi Uchida, Hitoshi Naito, Yuki Someya, Hiroki Tabata, Saori Kakehi, Tsubasa Tajima, Naoaki Ito, Satoshi Kadowaki, Yuya Nishida, Ryuzo Kawamori, Hirotaka Watada, Yoshifumi Tamura

**Affiliations:** Department of Metabolism & Endocrinology, Juntendo University Graduate School of Medicine, Tokyo 113-8421, Japan; Department of Metabolism & Endocrinology, Juntendo University Graduate School of Medicine, Tokyo 113-8421, Japan; Department of Metabolism & Endocrinology, Juntendo University Graduate School of Medicine, Tokyo 113-8421, Japan; Department of Metabolism & Endocrinology, Juntendo University Graduate School of Medicine, Tokyo 113-8421, Japan; Sportology Center, Juntendo University Graduate School of Medicine, Tokyo 113-8421, Japan; Sportology Center, Juntendo University Graduate School of Medicine, Tokyo 113-8421, Japan; Sportology Center, Juntendo University Graduate School of Medicine, Tokyo 113-8421, Japan; Department of Metabolism & Endocrinology, Juntendo University Graduate School of Medicine, Tokyo 113-8421, Japan; Department of Metabolism & Endocrinology, Juntendo University Graduate School of Medicine, Tokyo 113-8421, Japan; Department of Metabolism & Endocrinology, Juntendo University Graduate School of Medicine, Tokyo 113-8421, Japan; Department of Metabolism & Endocrinology, Juntendo University Graduate School of Medicine, Tokyo 113-8421, Japan; Department of Metabolism & Endocrinology, Juntendo University Graduate School of Medicine, Tokyo 113-8421, Japan; Sportology Center, Juntendo University Graduate School of Medicine, Tokyo 113-8421, Japan; Department of Metabolism & Endocrinology, Juntendo University Graduate School of Medicine, Tokyo 113-8421, Japan; Sportology Center, Juntendo University Graduate School of Medicine, Tokyo 113-8421, Japan; Department of Metabolism & Endocrinology, Juntendo University Graduate School of Medicine, Tokyo 113-8421, Japan; Sportology Center, Juntendo University Graduate School of Medicine, Tokyo 113-8421, Japan

**Keywords:** subclinical hypothyroidism, thyroid stimulating hormone, reference range, cerebral microbleeds, sarcopenia, older adults

## Abstract

**Context:**

Thyroid-stimulating hormone (TSH) levels physiologically increase with age. Applying a fixed adult reference range to older adults may cause overdiagnosis of subclinical hypothyroidism (SCH). This study examined whether adjusting the upper limit of the TSH reference range is appropriate by evaluating reference ranges and risks of geriatric conditions in older adults.

**Design and Setting:**

We analyzed 1626 community-dwelling adults aged 65-84 years from the Bunkyo Health Study. Reference ranges for TSH, free triiodothyronine (FT3), and free thyroxine (FT4) were calculated using the 2.5th-97.5th percentiles. Participants with normal FT4 were classified into 3 groups by TSH: euthyroid (0.61-4.23 mIU/L), mild SCH (>4.23 to <7.0 mIU/L), and moderate-to-severe SCH (≥7.0 mIU/L). Associations between TSH categories and geriatric conditions were evaluated using logistic regression.

**Results:**

With increasing age, TSH tended to increase, while FT3 decreased. The 2.5th-97.5th percentile range of TSH in the total cohort was 0.49-5.56 mIU/L. After adjusting for confounders, moderate-to-severe SCH was significantly associated with cerebral microbleeds (odds ratio [OR]: 4.22, 95% CI: 1.49-11.97) and sarcopenia (OR: 3.27, 95% CI: 1.02-10.52) compared to the euthyroid group. Mild SCH was not significantly associated with any geriatric condition.

**Conclusion:**

Age-related TSH elevation is common among older adults. Mild SCH was not linked to geriatric conditions, whereas moderate-to-severe SCH showed associations with cerebral microbleeds and sarcopenia. Given the limited number of participants in this category, these findings should be interpreted cautiously and require confirmation in longitudinal studies.

With global population aging, geriatric conditions such as dementia, sarcopenia or frailty, osteoporosis, and cardiovascular diseases have become major public health concerns. These conditions markedly affect quality of life and increase healthcare costs, underscoring the need for effective prevention and early detection strategies.

Among the physiological changes that accompany aging, alterations in thyroid function have received growing attention because of their potential effects on multiple organ systems [1]. Age-related changes in thyroid function often result in modest increases in thyroid-stimulating hormone (TSH) levels [2-5]. The National Health and Nutrition Examination Survey III reported that the upper reference limit of TSH increases with age, reaching approximately 7.49 mIU/L in individuals aged ≥80 years compared with the conventional threshold of 4.5 mIU/L [2].

In Japan, the current TSH reference range (0.61-4.23 mIU/L), established using individuals aged <60 years, may not reflect normal aging processes. This discrepancy could lead to overdiagnosis of subclinical hypothyroidism (SCH) and unnecessary treatment in older adults. Because Japan is an iodine-sufficient country, and TSH levels are generally higher in such populations, the applicability of prior findings remains uncertain, and epidemiologic data in this setting are limited [3, 6]. Although SCH has been associated with cardiovascular risk in some studies [7, 8], its relationship with other common geriatric conditions remains unclear. Understanding thyroid function in community-dwelling older adults in an iodine-sufficient country is therefore important, both to refine disease classification and to evaluate its clinical implications.

This study aimed to determine whether raising the upper limit of the TSH reference range is justified. Specifically, we investigated (1) the basic statistics and reference range of thyroid hormones in older adults and (2) the potential risks of geriatric conditions associated with SCH, as well as the TSH range within which such risks do not increase.

## Methods

### Study design and participants

This cross-sectional study used baseline data from the Bunkyo Health Study [9]. A total of 1629 community-dwelling individuals aged 65-84 years were recruited from Bunkyo-ku, an urban district in Tokyo, Japan, between October 15, 2015, and October 1, 2018. After excluding 3 participants with missing thyroid function data, 1626 participants remained in the analysis. Assessments were conducted over 2 days. On day one, cognitive function, muscle strength, and physical performance were evaluated. On day 2, following an overnight fast, body weight and composition were measured using bioelectrical impedance analysis (BIA), bone mineral density (BMD) was determined with dual-energy X-ray absorptiometry (DXA), and the Cardio–Ankle Vascular Index (CAVI) was assessed. Blood and urine samples were collected, and brain magnetic resonance imaging (MRI) was performed.

The study protocol was approved by the ethics committee of Juntendo University in September 2015 (initial approval No. 2015061; latest version No. M15-0057-M09). The study adhered to the principles of the Declaration of Helsinki. Written informed consent was obtained from all participants, who were informed of their right to withdraw at any time.

### Measurement of thyroid hormones

Fasting morning blood samples were analyzed by an external clinical laboratory (SRL Inc., Tokyo, Japan). Serum TSH, free triiodothyronine (FT3), and free thyroxine (FT4) levels were determined using electrochemiluminescence immunoassays. TSH concentrations were measured with the Elecsys TSH assay (Roche Diagnostics) on a Cobas 8000 e801 module (Hitachi High-Technologies). FT3 and FT4 levels were measured using Elecsys FT3 III and FT4 III assays (Roche Diagnostics), respectively, on the same analyzer. Anti-thyroid peroxidase antibody (anti-TPO Ab) and anti-thyroglobulin antibody (anti-Tg Ab) levels were determined using chemiluminescent enzyme immunoassays with the Lumipulse Presto TPOAb (Fujirebio Cat# 297148, RRID: AB_3738361) and TgAb (Fujirebio Cat# 297131, RRID: AB_3738360) assays on a Lumipulse L2400 analyzer (FUJIREBIO). Although international harmonization of TSH values has been implemented [10], a conversion factor of 1.0 was applied; thus, no adjustment was necessary.

To accurately assess age-related variations in thyroid function, individuals taking thyroid medications, steroids, or amiodarone; those positive for thyroid autoantibodies (anti-TPO Ab ≥ 5.3 IU/mL or anti-Tg Ab ≥ 19.3 IU/mL); and those with a personal or family history of thyroid disease were excluded, following the American Association for Clinical Chemistry guidelines [11]. For analyses examining SCH and geriatric conditions, participants using thyroid medications, steroids, or amiodarone, as well as those with hyperthyroidism or overt hypothyroidism, were excluded. The euthyroid group was defined as participants with both TSH and FT4 levels within the reference range. SCH was defined as elevated TSH (>4.23 mIU/L) with normal FT4 levels and classified as mild SCH (4.23 < TSH < 7.0 mIU/L) or moderate-to-severe SCH (TSH ≥ 7.0 mIU/L). The cutoff of 7.0 mIU/L for moderate-to-severe SCH was selected based on prior epidemiological evidence suggesting increased health risks at approximately this range [7, 12]. This threshold was used for observational risk stratification; treatment decisions generally follow guideline recommendations, with routine LT4 therapy typically considered when TSH is persistently ≥10 mIU/L [13].

### Measurement of cerebral small vessel disease

Whole-brain MRI was performed using a 0.3 T clinical MRI scanner (AIRIS Vento, Hitachi, Tokyo, Japan). The protocol included axial 3-dimensional (3D) time-of-flight magnetic resonance angiography (repetition time (TR), 35 ms; echo time (TE), 7.1 ms; and slice thickness, 1.2 mm), T2 *-weighted gradient echo (T2 *-WI) imaging (TR, 1000 ms; TE, 45 ms; flip angle, 20°; and slice thickness, 5 mm), and fluid-attenuated inversion recovery (FLAIR) imaging (TR, 11 000 ms; TE, 100 ms; inversion time (TI), 2000 ms; and slice thickness, 5 mm). The validity of the 0.3 T scanner was verified by comparing images obtained using both 0.3 T and 3 T scanners [14]. Cerebral microbleeds (CMBs) and lacunar infarctions were diagnosed by an experienced neuroradiologist blinded to all clinical data, using axial T2*-WI and FLAIR images.

### Measurement of BMD

BMD at the femoral neck was measured using DXA with the Discovery system (Hologic Inc., Marlborough, MA, USA) [15]. BMD values were expressed as T-scores, representing the number of SD from the mean BMD of a young reference population. Osteoporosis was defined according to World Health Organization criteria as a femoral neck T-score < −2.5 SD [16]. Current use of osteoporosis medications was also considered indicative of osteoporosis, regardless of T-score.

### Measurement of cognitive function

Cognitive function was evaluated using the Montreal Cognitive Assessment (MoCA) [17] and the Mini-Mental State Examination (MMSE) [18], each scored on a 30-point scale. The MoCA includes 9 items and the MMSE includes eleven. Mild cognitive impairment (MCI) was defined as a MoCA score ≤ 22 [19], and dementia as an MMSE score ≤ 23 [18].

### Measurement of handgrip strength and sarcopenia

Handgrip strength was measured in the standing position using a dynamometer (T.K.K. 5401; Takei Scientific Instruments, Niigata, Japan) [20]. Participants held the device at thigh level, and 2 measurements were obtained for each hand. The highest value for each hand was averaged for analysis. Body composition was assessed using BIA (InBody770, InBody Japan Inc., Tokyo, Japan). The skeletal muscle mass index (SMI) was calculated as appendicular skeletal muscle mass divided by height squared (kg/m²). Sarcopenia was defined according to the 2019 Asian Working Group for Sarcopenia criteria [21] as low handgrip strength (<28 kg for males, <18 kg for females) combined with low SMI (<7.0 kg/m² for males, <5.7 kg/m² for females).

### Other measurements

Nutritional status was assessed using the Brief-Type Self-Administered Diet History Questionnaire [22]. Hypertension was defined as systolic blood pressure ≥ 140 mmHg, diastolic blood pressure ≥ 90 mmHg, or current use of antihypertensive medication. Dyslipidemia was defined as low-density lipoprotein cholesterol ≥ 140 mg/dL, high-density lipoprotein cholesterol < 40 mg/dL, triglycerides ≥ 150 mg/dL, or current use of lipid-lowering medication. Diabetes was defined as fasting plasma glucose ≥ 126 mg/dL and/or, 2-hour glucose level ≥ 200 mg/dL during a 75-g oral glucose tolerance test (OGTT), and hemoglobin A1c (HbA1c) ≥ 6.5%, or current use of diabetes medication. Chronic kidney disease (CKD) was defined as an estimated glomerular filtration rate (eGFR) < 60 mL/min/1.73 m² or urinary albumin-to-creatinine ratio ≥ 30 mg/g creatinine [23]. Heart failure was defined as serum N-terminal pro–B-type natriuretic peptide (NT-proBNP) ≥ 400 pg/mL [24]. Arteriosclerosis was evaluated using the CAVI and the Ankle Brachial Index (ABI), both measured with an automatic waveform analyzer (Vascular Screening System VaSera VS-1500; Fukuda Denshi, Tokyo, Japan).

### Statistical analysis

Participants were categorized into 4 age groups: 65-69, 70-74, 75-79, and 80-84 years. Age-specific reference ranges for thyroid hormones were determined using the 2.5th-97.5th percentiles. Trends across age groups were examined using the Jonckheere–Terpstra trend test. Thyroid hormone and other continuous variables were expressed as mean ± SD or interquartile range. Categorical variables were expressed as percentages. Group differences were evaluated using 1-way analysis of variance (ANOVA) for continuous variables and chi-square tests for categorical variables. Homogeneity of variance was assessed using Levene's test. When variances were homogeneous, 1-way ANOVA with Bonferroni post hoc tests was used for pairwise comparisons; when variances were unequal, Welch's ANOVA with Games–Howell post hoc tests was applied. For comparisons of multiple geriatric conditions across SCH categories, *P* values from chi-square tests were adjusted for multiple testing using the Benjamini–Hochberg false discovery rate (FDR) procedure. Associations between SCH categories and geriatric conditions were analyzed using logistic regression. Three models were developed: Model 1 adjusted for age, sex, and body mass index; Model 2 additionally adjusted for alcohol intake and Brinkman index (cigarettes/day × years smoked); and Model 3 further adjusted for hypertension, diabetes, and dyslipidemia. All analyses were performed using IBM SPSS Statistics for Windows, version 30.0.0.0 (IBM Corp., Armonk, NY, USA). A 2-sided *P* value < .05 was considered statistically significant.

## Results

### Basic statistics and reference ranges for thyroid hormones

**Figure 1:**
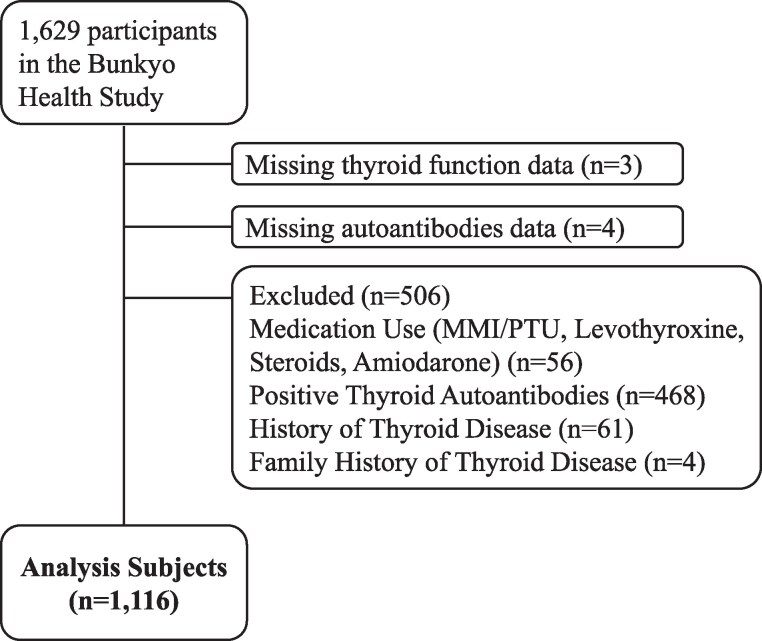
Patient flow chart of age-specific reference values for thyroid hormones.

After excluding participants using medications, those with positive thyroid autoantibodies, and individuals with a personal or family history of thyroid disease, 1116 participants were included in the reference range analysis (Fig. 1). Table 1 summarizes thyroid hormone levels by age group, along with age-specific reference ranges (2.5th-97.5th percentiles). TSH levels were significantly higher in the group aged 80-84 years compared with those aged 65-69 and 75-79 years. FT3 levels were significantly lower in the 80-84-year group than in all other age groups. Trend analysis using the Jonckheere–Terpstra test showed that TSH levels increased, whereas FT3 levels decreased, with advancing age. FT4 levels did not differ significantly among age groups and showed no age-related trend. Similarly, the upper limit of the TSH reference range rose with age, particularly among those aged ≥80 years. The overall 2.5-97.5th percentile range of TSH was 0.49-5.56 mIU/L.

**Table 1:** Thyroid hormone levels of participants by age group in the Bunkyo Health Study

		All	65-69 years	70-74 years	75-79 years	80-84 years	*P* value	*P* for trend
		*n* = 1116	*n* = 382	*n* = 310	*n* = 257	*n* = 167		
TSH (mIU/L)	Median (IQR)	1.69 (1.14-2.50)	1.64 (1.10-2.38)	1.70 (1.16-2.52)	1.69 (1.14-2.44)	1.84 (1.25-2.91)*^a,b^*	.018	.026
	2.5-97.5th percentiles	0.49-5.56	0.49-4.64	0.47-5.87	0.5-5.04	0.69-8.09	—	—
FT3 (pmol/L)	Mean ± SD	4.29 ± 0.49	4.35 ± 0.51	4.29 ± 0.46	4.27 ± 0.50	4.14 ± 0.46*^a,b,c^*	<.001	<.001
	2.5-97.5th percentiles	3.39-5.27	3.49-5.44	3.39-5.23	3.36-5.39	3.32-5.13	—	—
FT4 (pmol/L)	Mean ± SD	15.5 ± 1.97	15.6 ± 2.05	15.5 ± 1.83	15.5 ± 1.90	15.3 ± 2.17	.674	.120
	2.5-97.5th percentiles	11.7-19.6	11.7-19.6	12.0-19.2	11.8-19.8	11.2-20.3	—	—

*P* values are from one-way ANOVA (Welch's ANOVA if homogeneity of variances was not assumed). Post hoc comparisons were performed using Bonferroni or Games–Howell tests. Trends were tested using the Jonckheere–Terpstra test.

^*a*^<.05 vs 65-69 years.

^*b*^<.05 vs 75-79 years.

^*c*^<.05 vs 70-74 years.

### Group characteristics by TSH categories

**Figure 2:**
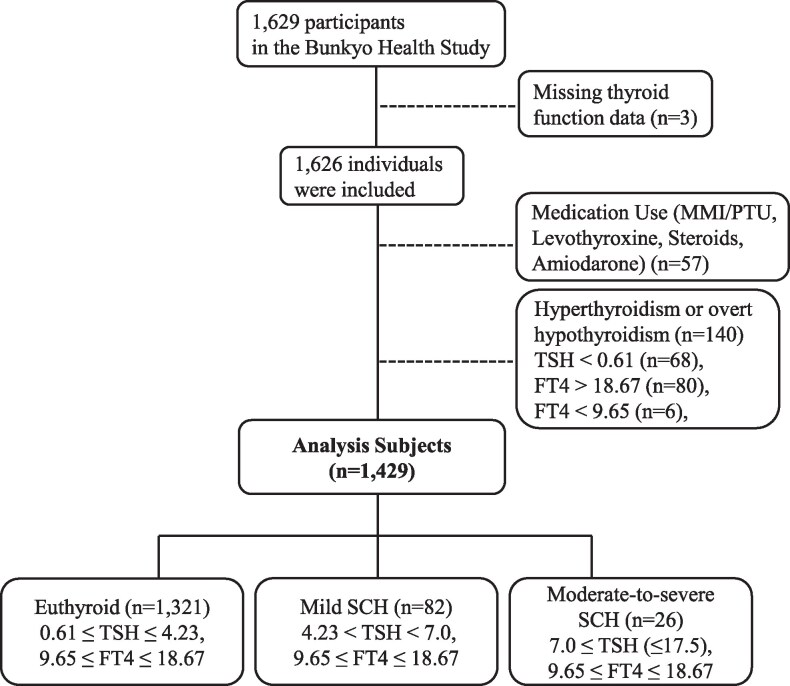
Patient flow chart of association between subclinical hypothyroidism and geriatric conditions.

After excluding participants taking thyroid medication, steroids, or amiodarone, and those diagnosed with hyperthyroidism or overt hypothyroidism, 1429 participants were included in the analysis of clinical associations with SCH. Participants were classified into 3 groups based on TSH levels: euthyroid (0.61 ≤ TSH ≤ 4.23 mIU/L, *n* = 1321), mild SCH (4.23 < TSH < 7.0 mIU/L, *n* = 82), and moderate-to-severe SCH (7.0 ≤ TSH ≤ 17.5 mIU/L, *n* = 26) (Fig. 2). Table 2 presents the characteristics of these groups. There were no significant differences in sex distribution among the groups. Although the overall ANOVA indicated significant age differences across the TSH categories, post hoc pairwise comparisons were not statistically significant, likely reflecting limited statistical power due to the small sample size in the moderate-to-severe SCH group. Body mass index and body fat percentage were similar across groups. Significant group differences were observed in FT3, FT4, and thyroid autoantibody positivity. Notably, 26.8% of participants in the euthyroid group were positive for thyroid autoantibodies. eGFR was significantly lower in both the mild and moderate-to-severe SCH groups than in the euthyroid group. In contrast, HbA1c, lipid profiles, and blood pressure did not differ significantly among groups.

**Table 2:** General baseline characteristics of participants by TSH level in the Bunkyo Health Study (*n* = 1429)

	Euthyroid	Mild SCH	Moderate-to-severe SCH	*P* value
	*n* = 1321	*n* = 82	*n* = 26	
Female	768 (58.1%)	47 (57.3%)	14 (53.8%)	.900
Age (years)	73.0 ± 5.3	74.5 ± 5.7	74.8 ± 5.6	.026
Body weight (kg)	57.1 ± 10.2	57.7 ± 9.8	56.2 ± 11.3	.786
Body mass index (kg/m^2^)	23.2 ± 3.0	23.4 ± 2.9	23.1 ± 3.4	.760
Percent body fat (%)	28.1 ± 7.2	29.6 ± 6.8	28.9 ± 6.6	.153
Alcohol intake (g)	13.6 ± 23.9	9.4 ± 18.4	8.2 ± 21.4	.155
Brinkman index	257 ± 492	250 ± 542	422 ± 711	.243
Energy intake (kcal/day)	1980 ± 606	1950 ± 557	1784 ± 521	.168
Systolic blood pressure (mmHg)	136 ± 17	138 ± 17	141 ± 17	.257
Diastolic blood pressure (mmHg)	84 ± 9.8	84 ± 10	88 ± 8.7	.081
CAVI	8.90 ± 1.07	8.87 ± .90	9.11 ± 1.03	.570
ABI	1.12 ± 0.25	1.06 ± 0.22	1.16 ± 0.22	.069
eGFR	67.2 ± 13.6	60.2 ± 14.5*^a^*	57.9 ± 12.6*^a^*	<.001
HbA1c (%)	5.82 ± 0.56	5.87 ± 0.55	5.90 ± 0.37	.522
TG (mg/dL)	99 ± 55	105 ± 65	97 ± 48	.691
T-Chol (mg/dL)	207 ± 37	206 ± 37	213 ± 32	.607
LDL-Chol (mg/dL)	122 ± 31	121 ± 34	128 ± 27	.582
HDL-Chol (mg/dL)	64 ± 16	62 ± 19	66 ± 18	.621
TSH (mIU/L)	1.87 ± 0.84	5.17 ± 0.68*^a^*	9.31 ± 2.64*^a,b^*	< .001
FT3 (pmol/L)	4.26 ± 0.47	4.19 ± 0.45	4.06 ± 0.46	.036
FT4 (pmol/L)	15.3 ± 1.62	14.1 ± 1.70*^a^*	13.1 ± 1.78*^a,b^*	<.001
Anti-TPO/Tg Ab positive	354 (26.8%)	30 (36.6%)	12 (46.2%)	.017

Data are presented as mean ± SD or *n* (%). *P* values were calculated by ANOVA (Welch's ANOVA if variances were unequal) for continuous variables and by chi-square test for categorical variables. Post hoc comparisons were performed using Bonferroni or Games–Howell tests.

Abbreviations: HDL-C, high-density lipoprotein cholesterol; LDL-C, low-density lipoprotein cholesterol.

^*a*^<.05 vs euthyroid.

^*b*^<.05 vs mild SCH.

### Association of SCH with geriatric conditions

We next examined the association between TSH categories and the prevalence of geriatric conditions. As shown in Table 3, significant differences were observed among groups in the prevalence of CMBs, low handgrip strength, sarcopenia, hypertension, and CKD. After adjustment for multiple comparisons using the FDR method, significant associations remained for cerebral microbleeds, low handgrip strength, sarcopenia, and chronic kidney disease, whereas the association with hypertension did not remain statistically significant. Logistic regression analyses were then performed for outcomes that remained significant after FDR correction to adjust for potential confounders, including age, sex, body mass index, alcohol intake, smoking, hypertension, dyslipidemia, and diabetes. As shown in Table 4, participants in the moderate-to-severe SCH group had significantly higher odds of CMBs (odds ratio [OR] 4.22, 95% CI: 1.49-11.97), low handgrip strength (OR 2.85, 95% CI: 1.18-6.90), and sarcopenia (OR 3.27, 95% CI: 1.02-10.52) compared with the euthyroid group. In contrast, moderate-to-severe SCH was not significantly associated with CKD after adjustment. No significant associations were found between mild SCH and any geriatric conditions.

**Table 3:** The prevalence of geriatric conditions by TSH level in the Bunkyo Health Study (*n* = 1429)

	Euthyroid	Mild SCH	Moderate-to-severe SCH	*P* value	q value (FDR)
	*n* = 1321	*n* = 82	*n* = 26		
Diabetes	165 (12.5%)	6 (7.3%)	2 (7.7%)	.297	0.446
Hypertension	850 (64.3%)	62 (75.6%)	21 (80.8%)	.028	0.084
Dyslipidemia	823 (62.3%)	58 (70.7%)	19 (73.1%)	.173	0.353
Cardiovascular disease	117 (8.9%)	8 (9.8%)	4 (15.4%)	.502	0.579
Ischemic heart disease	60 (4.5%)	5 (6.1%)	2 (7.7%)	.621	0.665
Dementia	41 (3.1%)	3 (3.7%)	3 (11.5%)	.057	0.143
Mild cognitive impairment	241 (18.2%)	18 (22.0%)	3 (11.5%)	.466	0.579
Heart failure	62 (4.7%)	7 (8.5%)	2 (7.7%)	.243	0.405
Chronic kidney disease	521 (39.4%)	43 (52.4%)	16 (61.5%)	.006	0.030
Osteoporosis	396 (30.0%)	28 (34.1%)	7 (26.9%)	.681	0.681
Low handgrip strength	242 (18.3%)	16 (19.5%)	11 (42.3%)	.008	0.030
Low skeletal muscle mass index	260 (19.7%)	12 (14.6%)	8 (30.8%)	.188	0.353
Sarcopenia	91 (6.9%)	2 (2.4%)	6 (23.1%)	.001	0.011
Cerebral microbleeds	63 (4.8%)	8 (9.8%)	5 (19.2%)	<.001	0.011
Lacunar infarction	229 (17.4%)	15 (18.3%)	7 (26.9%)	.447	0.579

Data are presented as *n* (%). *P* values were calculated by chi-square test. To account for multiple comparisons across outcomes, false discovery rate (FDR) correction was applied using the Benjamini–Hochberg procedure; FDR-adjusted q values are shown.

**Table 4:** Odds ratios (OR) with 95% CI for the associations between SCH and geriatric conditions by TSH level

Cerebral microbleeds	Prevalence (%)	Crude	Model 1	Model 2	Model 3
Euthyroid	4.8 (*n* = 63)	Reference	Reference	Reference	Reference
Mild SCH	9.8 (*n* = 8)	2.15 (0.99-4.65)	1.95 (0.89-4.27)	1.99 (0.90-4.36)	1.88 (0.85-4.14)
Moderate-to-severe SCH	19.2 (*n* = 5)	4.73 (1.73-12.95)	4.54 (1.62-12.68)	4.71 (1.67-13.24)	4.22 (1.49-11.97)
Sarcopenia					
Euthyroid	6.9 (*n* = 91)	Reference	Reference	Reference	Reference
Mild SCH	2.4 (*n* = 2)	0.34 (0.08-1.40)	0.37 (0.09-1.59)	0.36 (0.08-1.56)	0.34 (0.08-1.48)
Moderate-to-severe SCH	23.1 (*n* = 6)	4.06 (1.59-10.35)	3.46 (1.11-10.80)	3.55 (1.13-11.16)	3.27 (1.02-10.52)
Low handgrip strength					
Euthyroid	18.3 (*n* = 242)	Reference	Reference	Reference	Reference
Mild SCH	19.5 (*n* = 16)	1.08 (0.62-1.90)	0.90 (0.49-1.60)	0.88 (0.49-1.59)	0.91 (0.50-1.65)
Moderate-to-severe SCH	42.3 (*n* = 11)	3.27 (1.48-7.21)	2.79 (1.17-6.65)	2.79 (1.17-6.69)	2.85 (1.18-6.90)
Chronic kidney disease					
Euthyroid	39.4 (*n* = 521)	Reference	Reference	Reference	Reference
Mild SCH	52.4 (*n* = 43)	1.69 (1.08-2.65)	1.49 (0.93-2.38)	1.45 (0.91-2.32)	1.38 (0.86-2.22)
Moderate-to-severe SCH	61.5 (*n* = 16)	2.46 (1.11-5.46)	2.20 (0.96-5.05)	2.10 (0.91-4.83)	1.96 (0.85-4.57)

Data are presented as odds ratios (95% CIs) from logistic regression analyses. Three models were constructed: Model 1 adjusted for age, sex, and body mass index; Model 2 additionally adjusted for alcohol intake and Brinkman index (cigarettes/day × years smoked); and Model 3 further adjusted for hypertension, diabetes, and dyslipidemia.

In sensitivity analyses restricted to participants negative for thyroid autoantibodies, moderate-to-severe SCH remained associated with CMBs (OR 3.98, 95% CI: 1.00-15.78; *n* = 3/14) and low handgrip strength (OR 5.37, 95% CI: 1.63-17.71; n = 8/14), whereas the association with sarcopenia did not reach statistical significance (OR 3.05, 95% CI: 0.64-14.49; *n* = 3/14) in the fully adjusted model (Model 3).

## Discussion

This study provides new insight into age-related changes in thyroid function among community-dwelling older Japanese adults. Many participants had TSH levels exceeding the conventional adult reference range while remaining clinically euthyroid, consistent with prior findings that modest TSH elevation is common with aging. These results underscore the importance of accounting for physiological age-related variation when interpreting thyroid function in older adults [25]. More importantly, this study comprehensively examined the relationship between SCH and geriatric conditions. Mild SCH was not associated with increased risk, whereas moderate-to-severe SCH correlated significantly with higher risks of CMBs, low handgrip strength, and sarcopenia. Collectively, these findings suggest that adjusting the upper limit of the TSH reference range for older adults may reduce overdiagnosis and overtreatment. Furthermore, the health risks identified in participants with moderate-to-severe SCH (TSH ≥ 7.0 mIU/L) in this iodine-sufficient population were comparable to those in other regions, indicating that SCH-related adverse effects may be largely independent of iodine intake.

With global population aging, optimal management of SCH in older adults remains a key but unresolved issue. International guidelines typically recommend treatment when TSH persistently exceeds 10 mIU/L [26-29]. However, epidemiologic studies have suggested that TSH levels in the 7.0-9.9 mIU/L range may be associated with increased cardiovascular and cerebrovascular risk [7, 12], raising the possibility that clinically relevant risk may emerge below 10 mIU/L in selected individuals. Accordingly, we used 7.0 mIU/L as the cutoff for moderate-to-severe SCH for observational risk stratification. In our cohort, moderate-to-severe SCH showed associations with CMBs and sarcopenia, whereas mild SCH did not. These findings support the notion that mildly elevated TSH levels (approximately 5-6 mIU/L) may reflect physiological variation in older adults, while higher levels (≥7.0 mIU/L) may warrant closer clinical attention. Nonetheless, given the cross-sectional design and the small number of participants in the moderate-to-severe SCH group, these results should be interpreted cautiously and require confirmation in longitudinal and interventional studies.

Overt hypothyroidism and SCH have been associated with a hypocoagulable state, which may increase cerebral small vessel vulnerability [30, 31]. A cohort study of patients with minor stroke or TIA found that SCH was linked to a greater burden of cerebral small vessel disease [32]. In our cohort, lipid profiles, glucose tolerance, blood pressure, CAVI, ABI, smoking status, and body composition were comparable among groups, suggesting that nontraditional pathways may underlie the association between SCH and CMBs. The higher CMB prevalence among participants with elevated TSH aligns with prior observations and is particularly relevant in Japan, where cerebrovascular disease remains more prevalent than ischemic heart disease among older adults [33, 34]. CMBs predict future intracerebral hemorrhage and stroke [35], and a higher CMB burden has been linked to faster cognitive decline [36]. Thus, SCH may represent an underrecognized risk factor for cerebrovascular disease in older adults. Incorporating thyroid function assessment into cerebrovascular risk evaluation could be especially valuable in East Asian populations with a high prevalence of small vessel disease.

Participants in the moderate-to-severe SCH group exhibited a significantly higher prevalence of low handgrip strength and sarcopenia than those in the euthyroid group. Thyroid hormones are essential for skeletal muscle function through regulation of protein synthesis and mitochondrial activity, and hypothyroidism has been associated with muscle weakness and altered fiber composition [37]. Although evidence regarding SCH is limited, a study in older Chinese adults reported a higher prevalence of sarcopenia among individuals with SCH [38]. Proposed mechanisms include impaired protein synthesis, mitochondrial dysfunction, and chronic low-grade inflammation. Recent findings suggest that reduced FT3 and persistent inflammation may further promote muscle wasting [39]. These results indicate that SCH with TSH ≥ 7.0 mIU/L may negatively influence muscle function and contribute to frailty in older adults.

Regarding CKD, both mild and moderate-to-severe SCH groups demonstrated lower eGFR values than the euthyroid group (Table 3), consistent with prior studies linking SCH to reduced renal function [40]. However, CKD, defined by decreased eGFR or increased urinary albumin excretion, was not significantly associated with SCH after adjusting for potential confounders in logistic regression. Proposed mechanisms include reduced cardiac output, diminished renal perfusion, and increased vascular resistance [40]. Thus, while thyroid dysfunction may affect renal function, its impact in older adults likely reflects coexisting factors.

Therapeutically, several studies suggest that levothyroxine (LT4) therapy may slow eGFR decline in patients with both CKD and SCH [41]. Cardiovascular benefits have also been reported in selected groups, including patients with heart failure and reduced ejection fraction [42]. Nevertheless, evidence in older adults remains inconsistent. For instance, an observational UK study found fewer ischemic heart disease events in middle-aged SCH patients treated with LT4 but not in those aged ≥70 years [43]. Moreover, the TRUST trial and recent meta-analyses revealed no clear benefit of treating SCH in individuals aged ≥65 years [44-46]. These findings underscore the need for individualized treatment decisions that balance potential benefits and risks in older patients with SCH. However, limitations such as short follow-up, heterogeneous interventions, and variable treatment targets prevent definitive conclusions.

Strengths of this study include the use of a large community-based cohort of older adults, the inclusion of brain MRI in all participants for direct assessment of CMBs, and comprehensive evaluation of geriatric syndromes such as sarcopenia, cognitive impairment, and CKD. Thyroid autoantibodies were measured in all participants, allowing a more accurate assessment of age-related thyroid changes among antibody-negative individuals. However, this study has several limitations. Given the cross-sectional design, causality cannot be inferred and residual confounding may persist despite multivariable adjustment. In addition, the small size of the moderate-to-severe SCH subgroup (*n* = 26) limited statistical power and precision. Accordingly, these findings should be interpreted cautiously and require confirmation in longitudinal studies; treatment recommendations for individuals with TSH 7-9.9 mIU/L cannot be made based on the present results alone. Thyroid function was assessed only once at baseline. Because SCH can be transient, some degree of misclassification of thyroid status due to short-term fluctuations cannot be excluded. Repeated measurements would be needed to confirm persistent SCH and to better evaluate its long-term clinical implications. Furthermore, because thyroid ultrasonography was not performed, we were unable to assess structural thyroid abnormalities or directly evaluate the relationship between TSH levels and thyroid morphology. Finally, the generalizability of our findings may be limited because this study was conducted in a single, urban cohort of community-dwelling older Japanese adults from an iodine-sufficient region. Therefore, extrapolation to populations with different ethnic backgrounds, iodine intake, or healthcare settings should be made with caution.

## Conclusion

This study characterized age-related changes in thyroid function among community-dwelling older Japanese adults and found that moderate-to-severe SCH (TSH ≥ 7.0 mIU/L) was associated with CMBs and sarcopenia, whereas mild SCH showed no such relationship. These results suggest that mild TSH elevations up to approximately 5-6 mIU/L may reflect physiological aging, whereas levels ≥7.0 mIU/L may be associated with an increased likelihood of adverse health outcomes. Further longitudinal research is required to clarify the long-term health effects of SCH and to determine whether intervention at higher TSH levels improves outcomes. Until such evidence is available, management of subclinical hypothyroidism in older adults should remain individualized and conservative.

## Data Availability

Some or all datasets generated during and/or analyzed during the current study are not publicly available but are available from the corresponding author on reasonable request.

## Abbreviations

ABI: Ankle Brachial Index
ANOVA: analysis of variance
BIA: bioelectrical impedance analysis
BMD: bone mineral density
CAVI: Cardio–Ankle Vascular Index
CKD: chronic kidney disease
CMB: cerebral microbleed
DXA: dual-energy X-ray absorptiometry
eGFR: estimated glomerular filtration rate
FDR: false discovery rate
FT3: free triiodothyronine
FT4: free thyroxine
MCI: mild cognitive impairment
MMSE: Mini-Mental State Examination
MoCA: Montreal Cognitive Assessment
MRI: magnetic resonance imaging
OR: odds ratio
SCH: subclinical hypothyroidism
SMI: skeletal muscle mass index
TSH: thyroid-stimulating hormone
